# Long drives and red tape: mapping rural veteran access to primary care using causal-loop diagramming

**DOI:** 10.1186/s12913-022-08318-2

**Published:** 2022-08-23

**Authors:** Erin S. Kenzie, Mary Patzel, Erik Nelson, Travis Lovejoy, Sarah Ono, Melinda M. Davis

**Affiliations:** 1 Oregon Rural Practice-Based Research Network, Oregon Health & Science University, Portland, OR USA; 2 Independent Veteran Advocate, Portland, OR USA; 3 VA Office of Rural Health, Veterans Rural Health Resource Center, Portland, OR USA; 4 Center to Improve Veteran Involvement in Care, VA Portland Health Care System, Portland, OR USA; 5 Department of Psychiatry, Oregon Health & Science University, Portland, OR USA; 6 Department of Family Medicine and School of Public Health, Oregon Health & Science University, Portland, OR USA

**Keywords:** Causal-loop diagramming, Veteran, Systems science, Rural, Primary care, Health care access

## Abstract

**Background:**

Rural veterans experience more challenges than their urban peers in accessing primary care services, which can negatively impact their health and wellbeing. The factors driving this disparity are complex and involve patient, clinic, health system, community and policy influences. Federal policies over the last decade have relaxed requirements for some veterans to receive primary care services from community providers through their VA benefits, known as community care.

**Methods:**

We used a participatory systems mapping approach involving causal-loop diagramming to identify interrelationships between variables underlying challenges to veteran access to primary care and potential opportunities for change—known as leverage points in systems science. Our methods involved a secondary analysis of semi-structured qualitative interviews with rural veterans, VA staff, non-VA clinic staff and providers who serve rural veterans, and veteran service officers (VSOs) in the Northwest region of the US, followed by a two-part participatory modeling session with a study advisory board. We then applied Meadows’s leverage point framework to identify and categorize potential interventions to improve rural veteran access to primary care.

**Results:**

The final model illustrated challenges at the veteran, clinic, and system levels as experienced by stakeholders. Main components of the diagram pertained to the choice of VA or non-VA primary care, veteran satisfaction with the VA, enrollment in VA benefits and other insurance, community care authorization, reimbursement of non-VA care, referrals to specialty care, record sharing and communication between VA and non-VA providers, institutional stability of the VA, and staffing challenges. Fourteen interventions, including administrative and communications changes, were identified by analyzing the model using the leverage points framework.

**Conclusions:**

Our findings illustrate how challenges rural veterans face accessing health care are interconnected and persist despite recent changes to federal law pertaining to the VA health care system in recent years. Systems mapping and modeling approaches such as causal-loop diagramming have potential for engaging stakeholders and supporting intervention and implementation planning.

**Supplementary Information:**

The online version contains supplementary material available at 10.1186/s12913-022-08318-2.

## Background

One quarter of US veterans–4.7 million in total–live in rural areas and are more likely than their urban counterparts to be older, to be less financially secure, and to have more significant health needs that require more frequent, ongoing, and costly care [1]. Even with a network of over 800 VA Community Based Outpatient Clinics, access to primary care services for rural veterans remains a challenge for many [2]. Barriers such as transportation, inconsistent staffing, and administrative hurdles are well documented [3–8]. In recent years, the US Department of Veterans Affairs (VA) has introduced various policies to help improve access to care. The Choice Act of 2014 allowed VA benefits to be used at non-VA providers in some circumstances, known as *community care* [9]. In 2018, the Maintaining Internal Systems and Strengthening Integrated Outside Networks Act (MISSION Act) was signed into law in an effort to increase veteran access to care through a suite of policies and administrative changes, including reducing distance requirements for community care [10]. Under the MISSION Act, veterans are eligible to receive care from community providers if the VA services do not meet certain quality, availability, or access standards (e.g., less than 30-min average drive time for primary care), they are “grandfathered” in through the prior Choice Act, or it is in the veteran’s best medical interest [11, 12]. Veterans using community care report better access than veterans using VA primary care services, but lower quality communication and coordination [13].

A number of prior studies explore barriers in access to care based on individual stakeholder perspectives (e.g., patient, clinic) [3, 5–8]. However, due to the broad scope and size of the VA, policy changes related to care provision are complex and impact diverse stakeholders. Moreover, understanding the interrelationships between factors shaping rural veterans’ access to care is critical to identifying and adapting appropriate multilevel interventions for improving access [14]. To better understand current challenges following the passage of the MISSION Act and potential leverage points for change, we used a participatory systems modeling approach called *causal-loop diagramming* to synthesize stakeholder perspectives of rural veteran access to primary care [15]. We then analyzed this model to identify potential interventions to improve access. We present the modeling process and outcomes and discuss the advantages and limitations of this approach for developing multilevel interventions.

## Methods

**Fig. 1:**
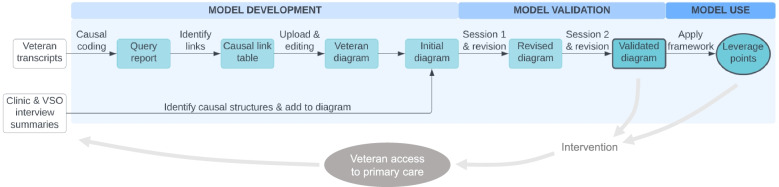
Process of model development, validation, and use. Study activities in the blue shaded area are shown in the broader research context. Data gathered in prior interviews was analyzed for causal structure and used to generate a draft causal-loop diagram. The diagram was validated in two virtual sessions with a stakeholder panel and used to identify potential leverage points for improving rural veterans’ access to primary care. The validated diagram and leverage point analysis will be used in future research to pilot an intervention, which will be qualitatively evaluated. Future rounds of modeling could inform intervention refinement and scale-up

Our team used causal-loop diagramming to synthesize stakeholder perspectives about complex dynamics underlying rural veteran access to primary care in Oregon, Washington, and Idaho. We then used the resulting model to categorize leverage points for improving access to care. Figure 1 provides an overview of the process we used.

### Causal-loop diagramming

**Fig. 2:**
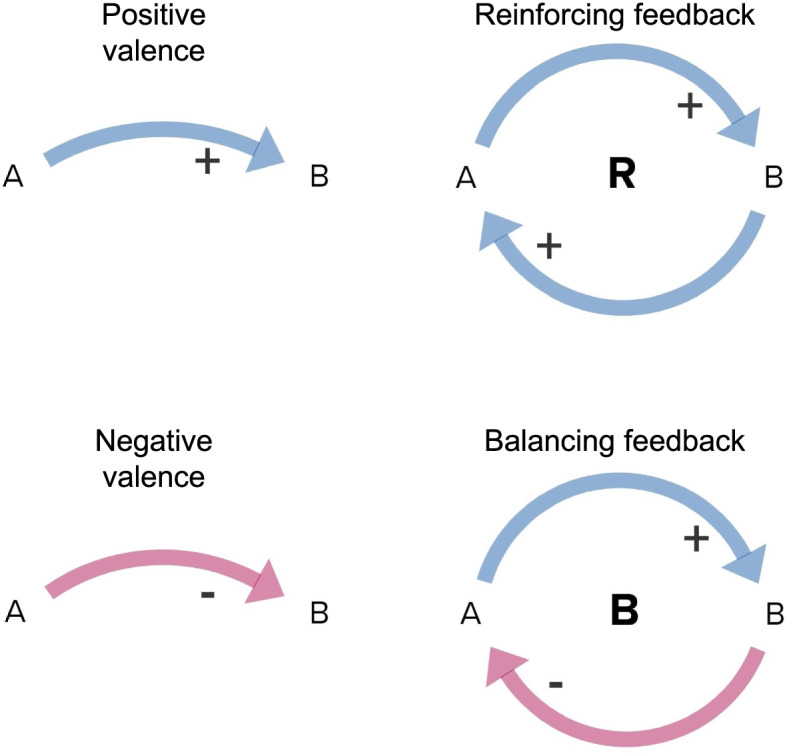
Causal-loop diagram notation. In causal-loop diagrams, causal links between variables have a positive or negative valence corresponding to whether the second variable increases or decreases in a way that is the same as or opposite to the first variable. Feedback loops are configurations of causal links that display circular logic and can be reinforcing (exponential behavior) or balancing (trend toward a set point)

Causal-loop diagramming originated in the field of system dynamics as a way to facilitate the development of computational models and teach feedback dynamics [15]. It has since become a standalone method for describing complex interactions between variables in a system [16, 17]. A key feature of this method is the identification of feedback loops, which are the source of nonlinear behavior in complex systems [15]. Causal-loop diagrams consist of variables and relationships between them represented in a node-and-edge format, as illustrated in Fig. 2.

Causal-loop diagrams reflect the mental model, or perspective, of the person or people involved in their development [15]. The relationships described in the model are best understood as hypothesized, assumed, or believed by the modelers or participants. The use of the word *causal* refers to the directionality of the relationship between two variables as understood by the people whose perspective is being described in the diagram and does not imply formal causal inference. This causal notation allows for communication about how variables are thought to affect one another. Participatory systems mapping using causal-loop diagramming is a qualitative method, although inclusion of specific links or structures can be supported by qualitative or quantitative evidence. Certain common configurations of causal links are called *archetypes* [15].

### Model development

To develop and validate the causal-loop diagram, we used an iterative, participatory approach [17, 18] involving our study team and stakeholders with an interest in rural veteran access to care. An initial model reflecting the results of qualitative interviews with stakeholders (veterans, clinic and VA staff) was validated and refined through a participatory session including interviewees and additional stakeholders from our study’s Rural Veterans Advisory Board. The aim of the model was to visually describe the complex relationships between factors underlying rural veterans’ access to primary care as understood by stakeholders. Our scope related to veterans residing in rural areas of Oregon, Washington and Idaho—all Northwest states within Veterans Integrated Services Network 20 (VISN 20) [19]. We use *rural veteran* and *veteran* interchangeably to refer to our study population. Model development was led by an analyst trained in systems modeling and supported by study team members with expertise in qualitative research, veteran health care, and health care access.

For the initial model, we conducted a secondary analysis on transcripts from semi-structured qualitative interviews conducted as part of a needs assessment to support the identification of interventions to improve rural veterans’ access to primary care. The interviews had been previously conducted with rural veterans (*n* = 13), veteran service officers (VSOs; *n* = 12), and clinicians and staff from non-VA clinics (*n* = 13), and VA clinicians and administrative staff (*n* = 3) between May and September 2020. Participants were recruited until the point of saturation, when no new relevant information was obtained during the interviews [20]. The purpose of these interviews was to identify barriers to rural veteran access to care across patient, clinical, and institutional levels. We used causal-loop diagramming as a way to integrate and compare qualitative findings across stakeholder groups and as a platform for participatory engagement and refinement of understanding. This study was approved by the Veterans Affairs Portland Health Care System (VAPORHCS) and Oregon Health & Science University (OHSU) joint IRB (eIRB#20,843).

#### Interview analysis

Causal relationships were identified in source data in two ways: veteran transcripts were first coded directly for causal information using a process outlined in prior research by our team [17], while previously compiled qualitative summaries of clinic and VSO data were used to incorporate those stakeholder perspectives. To reflect prioritization of veterans’ experience in the model, we based the initial draft on the analysis of the veteran interviews.

Each veteran transcript was reviewed in ATLAS.ti to identify causal structures, including causal links, feedback loops, and archetypes [17]. When a causal structure was identified, a code and note were attached to the corresponding quotation in the software (e.g., code: causal link; note: VA provider turnover (-) Veteran satisfaction with VA care). After all transcripts were coded, we ran a query using the codes related to causal structure (i.e., causal link, feedback loop, and archetype) to compile all relevant quotations. Quotations were compiled by participant rather than analyzed by individual participant because the aim was to develop a model that synthesized stakeholder perspectives [21]. The query report was then reviewed and all causal structures were entered into tables listing variables to be included in the model and causal links connecting them. Categories corresponding to codes used in the prior qualitative coding as well as some emergent categories were included as tags associated with variables and connections. The spreadsheet containing the tables was then uploaded to Kumu, a web-based visualization platform [22]. We then arranged the layout of the diagram to increase readability and edited the diagram to reduce repetition and connect related sub-models and links. This editing by the modeling team is a standard practice in systems diagramming [15] that we made more transparent by associating tags with causal links imputed by the modeling team.

For qualitative data that had already been summarized and thematically analyzed [23] by our team for an internal funder report (clinic and VSO participants’ data only), summaries describing main themes were then used to identify causal structures. These structures were compared to the draft model and unique structures were added. The data sources (veteran, non-VA clinic, VA health system informant, VSO and modeler) supporting each variable and causal link were tracked in the model. An initial model draft was iteratively revised through a series of meetings with the study team. Model structure adhered to established standards for causal-loop diagramming illustrated in Fig. 2 [15].

#### Model visualization

We then created selective displays of the model variables and relationships by stakeholder type using the interactive features of Kumu. To help “tell the story,” we added interview quotations and supplemental information to some key variables and feedback loops. The modeling team identified sub-models or regions within the model reflecting challenges veterans face accessing care. Finally, we created a Kumu presentation that walks the viewer step by step through the model alongside descriptive text.

### Model validation through stakeholder engagement

The initial model draft was validated through a two-part participatory session with our Rural Veteran Advisory Board and additional stakeholders, including veterans, VSOs, VA staff and clinicians, and non-VA clinicians and clinic staff. The primary purpose of these sessions was to share findings from the needs assessment and facilitate a dialogue to inform the prioritization of potential interventions. We presented the model draft to communicate findings; the subsequent discussion of the model provided an opportunity to validate and refine the model.

#### Validation participant characteristics

A total of 13 stakeholders participated in the two modeling sessions; 11 in the first and 9 in the second; see Table 1. Eleven participants were recruited from our Rural Veterans Advisory Board, which was convened in 2019 to provide strategic guidance to Veteran and VA focused research projects conducted by the Oregon Rural Practice-based Research Network (ORPRN). Board members are nominated with the aim of forming a working advisory group informed by diverse service, VA or healthcare experiences, geographic location and area of expertise. Participants include veterans, VA staff and clinicians, non-VA staff and clinicians that serve rural veterans, and VSOs. All participants lived in VISN 20. Two additional participants were recruited from our interview participants to ensure representation from all stakeholder groups during model validation.

**Table 1:** Roles held by participatory session participants

Stakeholder Roles	No. of participants	
	**1st session**	**2nd session**
Veteran	5	4
Veteran service officer	3	2
VA primary care provider	2	0
Non-VA primary care provider	2	3
Non-VA primary care staff member	1	1
VA administrative staff	1	1
VA funder representative	1	1
Total individuals	11	9

Some veteran participants also held professional roles working in VA or non-VA healthcare or worked as VSOs. At least one care provider had a close family member who was a veteran. Ten session participants had previously been interviewed for the study and were invited to participate given their interest in the study, stakeholder role and insights. Participants were largely consistent between the two sessions, with 7 participants attending both meetings.

#### Modeling validation approach

Two 120-min sessions were held over videoconference using Zoom in April 2021. The sessions were recorded and one qualitative analyst took field notes. At the first session, the lead modeler presented the model using a version of the Kumu presentation that was optimized for a videoconference format with less text and larger diagrams. The discussion was facilitated by the lead modeler, the project participants then provided feedback on the model, including sub-models and structures that reflected their understanding and aspects of rural veterans’ access to health care they did not feel were adequately represented in the model. The model was used as a springboard for discussion of the challenges of rural veteran access to care.

At the second session conducted two weeks later, the research team summarized the main themes from the first session and potential priority areas for intervention development. Participants were encouraged to share the group model with colleagues between sessions and reported on feedback collected in the interim. The discussion was facilitated by the project manager and principal investigator. Participants provided feedback on potential interventions and what successful outcomes might look like. Following the participatory sessions, the lead modeler reviewed the video recording and field notes to identify necessary changes to the model and made the modifications in Kumu.

### Model analysis and use

#### Description of model features

Several types of model features were identified in the final diagram: reinforcing and balancing feedback loops, exogenous drivers, hubs, and archetypes. *Feedback loops* are the source of nonlinear behavior in complex systems and constitute a key aspect of system structure. *Exogenous drivers* are variables that affect another variable, but are not themselves affected by another variable in the model [24]; in other words, they are connected to outgoing but not incoming causal links. Hubs or hub-and-spoke formations are defined by the number of causal links going in or out from a single variable. In casual-loop diagramming, exogenous drivers represent system boundaries because they represent places at which precipitating factors have been determined to be outside the scope of the model [15]. Although not commonly identified in causal-loop diagrams, *hubs* are a feature of network mapping that illustrate variables that play a central role in information transfer in systems [25]. In causal models, hubs are variables that connect sub-models or interface with multiple exogenous drivers. Identification of hubs can draw attention to variables in the model that have outsize influence over the behavior of the system, even if not embedded in a feedback loop, due to their position in the causal structure [26, 27]. In contrast with network mapping, which quantitatively analyzes and compares hubs, causal-loop diagramming can highlight hubs for the purpose of drawing attention to influential parts of the model that might be missed if only feedback loops are examined. For the purpose of this analysis, we defined hubs as variables in the upper decile of link density [26]. *Archetypes* are certain configurations of variables and causal links that communicate common situations through similar causal structures [15]. We identified them by comparing the complete model with Kim’s list of common archetypes [28].

#### Application of Meadows’s leverage point hierarchy

**Fig. 3:**
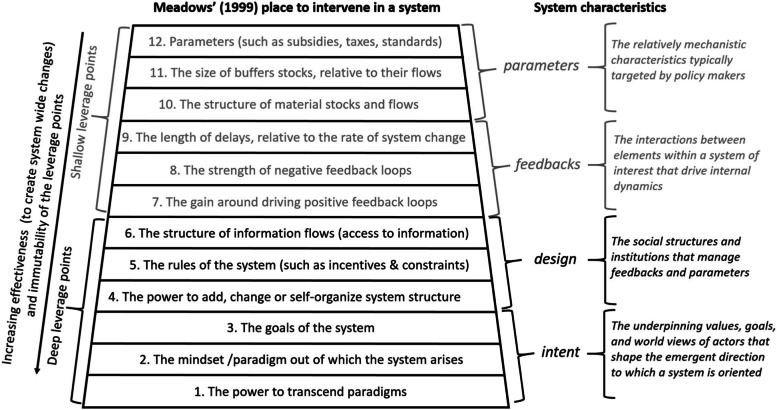
Meadows’s places to intervene in a system, reproduced with permission from Abson et al. 2017 [30]

Meadows’s hierarchy of potential leverage points is a well-known framework within systems science [29], which is shown in Fig. 3. To inform prioritization of potential interventions that address rural veteran access to primary care, our study team applied Meadows’s framework to the causal-loop diagram and subsequent discussion of leverage points.

In Meadows’s framework, leverage points are ranked according to their capacity to affect change and their difficulty to change, as seen in Fig. 3. Leverage points toward the top of the diagram relate to minor changes to policies or procedures that might change limits or quantities but not transform the structure of a system (e.g., the MISSION Act changing the distance requirements for accessing community care). Leverage points toward the bottom of the diagram indicate degrees of system redesign and transformation (e.g., a shift to a single-payer system).

## Results

### Diagram overview

**Fig. 4:**
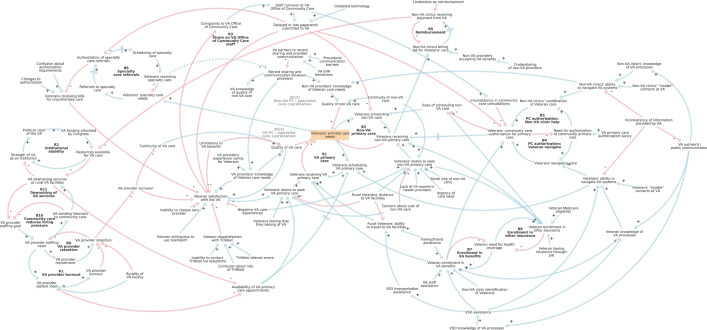
Causal-loop diagram of rural veteran access to care. Arrows indicate hypothesized causal relationships in stakeholder mental models as gleaned from secondary analysis of semi-structured qualitative interviews and participatory modeling sessions. Blue arrows have a positive valence, while red arrows have a negative valence. Sub-models include choice of VA or non-VA primary care, veteran satisfaction with the VA, enrollment in VA benefits and other insurance, community care authorization, reimbursement of non-VA care, referrals to specialty care, record sharing and communication between VA and non-VA providers, institutional stability of the VA, and staffing challenges. A web-based walkthrough of this diagram is available at https://ekenzie.kumu.io/caravan-rural-veteran-access-to-primary-care-stakeholder-interviews-causal-loop-diagram

Our final causal-loop diagram includes 94 variables, 144 causal links, and 121 feedback loops, as shown in Fig. 4. Sub-models or regions of the model include choice of VA or non-VA primary care, veteran satisfaction with the VA, enrollment in VA benefits and other insurance, community care authorization, reimbursement of non-VA care, referrals to specialty care, record sharing and communication between VA and non-VA providers, institutional stability of the VA, and staffing challenges.

An interactive web-based walkthrough of the diagram showed in Fig. 4 was developed and has been made publicly available to accompany this publication.^1^ This online version enables selective display of results by stakeholder type (veteran, VA, non-VA, VSO). We found there was a good deal of agreement between the mental models of veteran, non-VA, VA, and VSO stakeholders. Notably, the map components were nearly identical between veteran and VSO informants. Non-VA providers and clinic staff also had a good degree of overlap with veterans and VSOs, with the addition of administrative challenges of reimbursement and record sharing. The VA health system informants contributed an insider perspective on staffing and other factors driving challenges at the VA. Key features of the diagram identified during analysis—feedback loops, hubs, and exogenous drivers—are featured in Table 2.

**Table 2:** Key components of causal-loop diagram of rural veteran access to care

Type	Component
Reinforcing feedback loops	R1: VA provider burnout R2: Institutional stability R3: Strain on VA Office of Community Care staff
Balancing feedback loops	B1: VA primary care B2: Non-VA primary care B3: PC authorization: Non-VA clinic help B4: PC authorization: veterans navigate B5: Reimbursement B6: Specialty care referrals B7: Enrollment in VA benefits B8: Enrollment in other insurance B9: VA provider retention B10: Community care reduces hiring pressure B11: Downsizing of VA services B12: Non-VA PC: specialist care coordination B13: VA PC: specialist care coordination
Hubs (number of causal links in parentheses)	Veteran satisfaction with the VA (12); Veteran enrollment in VA benefits (9); Veterans’ desire to seek non-VA primary care (9); Veterans’ community care authorization for primary care (8); Veteran enrollment in other insurance (7); Veterans’ primary care needs (7); Delayed or lost paperwork submitted to the VA (6); Veterans’ ability to navigate VA systems (6); Veterans’ desire to seek VA primary care (6); Veterans receiving non-VA primary care (6)
Exogenous drivers	Changes to authorization; Concern about cost of non-VA care; Confusion about authorization requirements; Confusion about role of TriWest; Credentialing of non-VA providers; Family/friend assistance; Inability to choose own provider; Inability to contact TriWest by telephone; Inconsistency of information provided by VA; Lack of VA women’s health providers; Limitations on reimbursement; Limitations to VA benefits; Negative VA care experiences; Non-VA clinic identification of veterans; Non-VA clinics’ “insider” contacts at VA; Outdated technology; Procedural communication barriers; Rural veterans’ distance to VA facilities; Rurality of VA facility; Small size of non-VA clinic; TriWest referral errors; Urgency of care need; VA EHR limitations; VA primary care authorization policy; VA providers’ experience caring for veterans; VA staff assistance; Veteran having insurance through job; Veteran Medicare eligibility; Veteran willingness to use telehealth; Veterans feeling that they belong at the VA; Veterans’ “insider” contacts at VA

*Abbreviations*: *EHR* electronic health records, *PC* primary care, *VA* Veterans Administration

The reinforcing loops listed in Table 2 describe dynamics which compound or exacerbate themselves. Notably, two of the three reinforcing loops included in the model describe VA staff burnout. The balancing loops all describe a need that is filled or needs to be filled: primary or specialty care needs (B1, B2, B6), authorization of primary care (B3, B4), reimbursement (B5), enrollment in health coverage (B7, B8), staffing (B9, B10), facilities (B11), and communication (B12, B13).

All of the hubs listed in Table 2 describe variables that affect multiple parts of the diagram. The upper decile of link density for this model was six or more causal links, so hubs were determined using that definition. Delayed or lost paperwork, for example, can impact veterans’ care in multiple ways. Similarly, if veterans are not skilled at navigating VA care, they can encounter many kinds of challenges as they seek care. Finally, the exogenous drivers included in Table 2 reflect VA policies (e.g., limitations on reimbursement, VA primary care authorization policy) and characteristics (e.g., *Rurality of VA facility*); veteran characteristics, such as social support or connections (e.g., *family/friend assistance*, *veteran “insider” contacts at VA*), attitudes (e.g., *veteran willingness to use telehealth*), and other contextual factors (e.g., *veteran Medicare eligibility*); and characteristics and behaviors of non-VA clinic staff and providers (e.g., *Non-VA clinics’ identification of veterans*). Some variables included as exogenous drivers, such as *veterans feeling that they belong at the VA*, could have been made endogenous by connecting them to causes such as *Negative VA care experiences*, but were made exogenous by the modeling team out of a need for parsimony. In causal-loop diagramming, it is part of the modeling team’s role to make decisions about model boundaries [15].

#### Choice of primary care provider

In the center of the diagram, feedback loops B1 and B2 show that veterans receive primary care from VA or non-VA providers, depending on their distance from VA providers, type of health benefits or insurance they use, preferences, concerns, and prior experience. Rural veterans who live at least 30-min driving distance from a VA primary care provider are able to use their VA benefits to receive care from non-VA providers (“community care”), as determined by the MISSION Act of 2018 [10]. Veterans participating in Medicare or private insurance often use that coverage when seeking care from non-VA providers due to convenience, even though they often face higher costs than with VA community care.

#### Satisfaction with the VA

Veterans’ attitudes toward the VA as an institution as well as prior VA health care experiences shape their satisfaction with the VA and likelihood of pursuing VA health care (connections surrounding *veteran satisfaction with the VA* in Fig. 4). Veteran interview participants generally felt that VA providers' experience caring for veterans made them more knowledgeable about veteran needs. However, veterans were frustrated by experiences in which specific medications, equipment, or services (e.g., updated oxygen tanks) were not covered by the VA, processes for getting care covered were confusing or delayed, or VA providers gave substandard care. Veterans were also largely dissatisfied with TriWest, the company contracted by the VA to coordinate aspects of community care in VISN 20. Veterans expressed frustration with frequent turnover of VA providers, which compromised continuity of care. This was particularly difficult for people with traumatic or complicated medical histories. Provider turnover was described as more of a challenge at rural VA facilities than at urban VA facilities.

#### Drifting goals for VA provider staffing

**Fig. 5:**
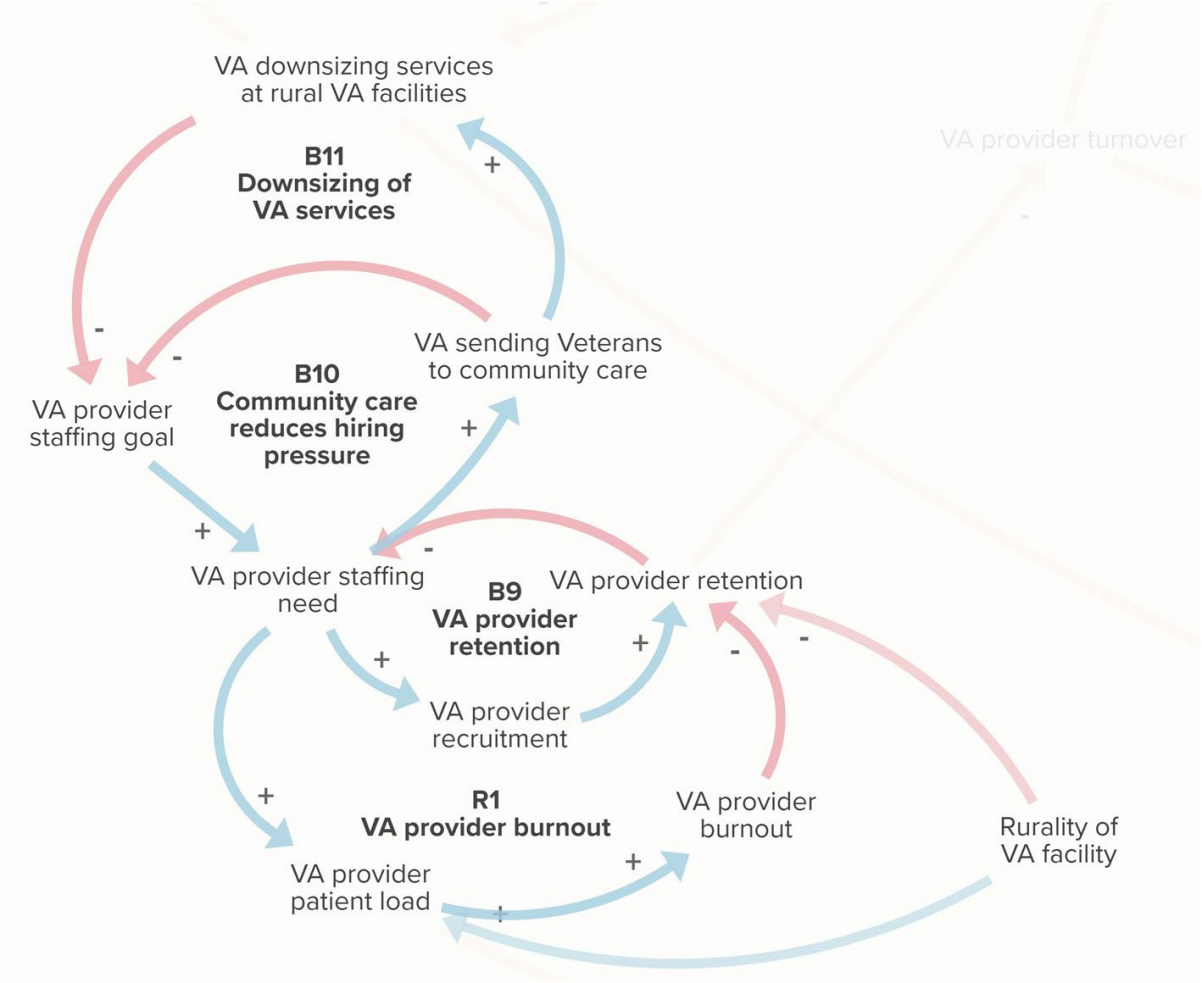
Drifting goals archetype for VA provider staffing. Loop R1 is a reinforcing loop illustrating how high patient loads per provider leads to burnout and negatively impacts provider retention, which undermines efforts to recruit and retain providers to meet staffing needs (loop B9). Rurality further impacts patient load and difficulty of retention. Veterans expressed concern that by sending veterans to community care, the VA would downsize their own VA facilities (loop B11) and make less effort to hire their own providers (loop B10)

Loop B9 is a goal-directed balancing feedback loop that shows that through recruitment and retention efforts, staffing needs are brought into line with staffing goals. Loop R1 is a reinforcing feedback loop, which describes a 'vicious cycle' in which provider retention and burnout amplify each other over time. Loop B10 describes how sending veterans to community care reduces hiring pressure by changing the VA staffing goal. This configuration of loops R1, B9, and B10 constitute a ‘drifting goals’ systems archetype [31], as illustrated in Fig. 5. In the drifting goals archetype, ambitious or unattainable goals are adjusted when an alternative way of addressing the underlying need is met [29]. Loop B11, which describes veterans’ concerns that the community care program drives downsizing at VA facilities, exacerbates the drifting goals effect.

#### Impact of community care on institutional stability

The strength of the VA as an institution depends in part on resources allocated by Congress. The VA and other veteran-serving organizations lobby Congress to prioritize funding for the VA. Funding of the VA supports better access to VA health services as well as further enables effective lobbying. Loop R2 in Fig. 4 is a reinforcing loop that describes this scenario. Increased resources lead to greater ability to obtain funding, while conversely, reduced resources weaken the strength of the VA and its ability to lobby for funding. Because the amount of money allocated by Congress is finite, reimbursements for community care take resources outside of the VA system. Some of our informants expressed frustration with VA community care based on the idea that it weakens the VA.

#### Community care authorizations and challenges navigating VA

Balancing loops B3 and B4 describe how veterans and non-VA clinic staff navigate the community care authorization process. Inconsistent information from the VA complicates the process. Both veterans and non-VA clinic staff said that it was helpful to have an ‘insider’ contact, someone that they personally knew at the VA to help them navigate the community care authorization process. These relationships are often temporary, however, due to staff turnover.

#### Delayed or lost paperwork

Many interviewees decried delayed or lost paperwork submitted to the VA, which affects many processes such as community care authorizations, scheduling of care, and specialty referrals (as shown in connections from the *delayed or lost paperwork submitted to the VA* variable at the top of the diagram in Fig. 4). VA health system informants cited staffing issues and outdated technology, such as reliance on fax machines, as a source of delays and lost paperwork. These disruptions caused significant frustration on the part of veterans and non-VA clinic staff and clinicians.

#### Administrative challenges for community care providers

To be eligible to bill the VA for health services, non-VA clinics must go through a complicated and often changing credentialing process that poses an administrative hurdle for smaller clinics, according to interviewees. Reimbursement for services (loop B5 in Fig. 4) is typically lower from the VA than from private insurance. Referrals to specialty care (loop B6) require their own authorizations and are often delayed. When authorization for care is not approved, veterans can receive unexpected bills for care.

#### Record sharing and communication between providers

One of the most common complaints from non-VA and VA providers and clinic staff was the lack of record sharing and communication processes between providers in different health systems. In Fig. 4, the connections surrounding the variable *record sharing between providers* illustrates these challenges. Balancing loops B12 and B13, indicated in gray text in Fig. 4, illustrate loops that could be strengthened if record sharing between providers was improved (as shown with the dotted line between *veterans receiving specialty care* and *record sharing between providers*).

### Model validation

#### Stakeholder appraisal of model

In the validation sessions, stakeholders conveyed a positive impression of the model. One VSO said, “I love it,” while a VA participant said we “hit the nail on the head.” The participants expressed support for the inclusion of the existing sub-models and variables. None of the stakeholders disagreed with the claims described in the model or suggested deleting a variable or relationship.

#### Summary of model changes

Stakeholder participants suggested a variety of additions and clarifications to the model during the first session, and two participants sent further suggestions in writing following the meeting. After the conclusion of the stakeholder process, we reviewed the recording and written suggestions and incorporated them into the diagram to produce a “version 2.0.” This version was then circulated to participants. These recommendations added further detail and did not significantly change model structure. In all, 8 variables, 15 connections, and one named feedback loop were added to the model. The feedback was also used to inform the inclusion of text attached to variables in the web-based model version. All model changes made during validation are included in Fig. 4; more information about these changes is available in Additional File 1.

### Identification of leverage points

Following the model validation and intervention brainstorming conversations with stakeholders, we categorized possible interventions identified using the model according to Meadows’s framework. Table 3 shows the results of this analysis, which includes aspects of the MISSION Act for context.

**Table 3:** Potential leverage points to improve rural veteran access to primary care

Meadows’s Places to Intervene	Interventions
12. Parameters (e.g., subsidies, taxes, standards)	Change distance requirements for community care (MISSION Act); broaden benefits eligibility (e.g., service connection, disability)
11. Buffers	Increasing staffing at VA Office of Community Care (weaken loop R3)
10. Material stocks and flows	Improving technological aspects of communication (switching from fax machines)
9. Delays	Extend time between primary care authorization renewals (add delays in loops B3-B4); reduce delays for specialty care authorizations and prescriptions (loop B6); reduce appointment delays through evening and weekend availability (loops B1-B2)
8. Balancing feedback loops	Improving record sharing and provider communication (strengthen loops B12-B13); Veteran identification in non-VA clinics (strengthen loop B7)
7. Reinforcing feedback loops	Reduce VA provider burnout and improve retention to improve continuity of VA care (weaken loop R1; strengthen B9)
6. Information flows	Improve VA communication to other stakeholders about processes (strengthen loops B3-B4); improve online systems for tracking care (referrals, authorizations) or communicating with providers (strengthen loops B6, B12-B13); improve communication between veterans and non-VA, VSO, community; gather & communicate data about wait times between VA & non-VA providers
5. Rules (e.g., incentives, constraints)	Change VA cost of living adjustment disincentivizing rural staff and providers (strengthen loop B9); increase VA budget for veteran healthcare (strengthen loop R2); allow VA benefits to function as secondary insurance
4. Self-organization	Reduce administrative barriers to process redesign and adaptation at the VA; offer case management or navigation services as workarounds (e.g., community health workers, through VSOs); form community coalitions
3. Goals	Change VA’s conflicting incentives between improving access to care and keeping funding inside VA
2. Mindset / paradigm	Single-payer systems (e.g., Australian gold card)
1. Transcending paradigms	None

## Discussion

As part of a needs assessment to inform multilevel interventions to improve rural veteran access to primary care, we used a participatory systems mapping approach involving causal-loop diagramming to present an integrated perspective of the complex dynamics driving persistent challenges facing rural Veterans. Our study integrates findings across multiple stakeholder groups and describes complex interrelationships between barriers and other causal factors shaping veterans’ experience of and access to care, including social, political, economic, technological, personal, and administrative dynamics. Primary sub-sections of the model include choice of VA or non-VA primary care, veteran satisfaction with the VA, enrollment in VA benefits and other insurance, community care authorization, reimbursement of non-VA care, referrals to specialty care, record sharing and communication between VA and non-VA providers, institutional stability of the VA, and staffing challenges. By applying Meadows’s framework, we categorized leverage points identified during the stakeholder process and subsequent study team discussions. Interventions with the greatest potential leverage involve structural changes to the VA health system. By illuminating interconnections between multilevel factors, this work has the potential to guide future research and efforts to improve veteran access to care.

### Findings in context

Our findings largely align with prior research into the barriers faced by rural veterans in accessing health care. Numerous instruments have been designed to assess access and care coordination relevant to cross-system care [32]. Prior findings illustrate challenges community care providers experience delivering care to Veterans. Our findings highlight some of these challenges and include community care providers’ limited knowledge of military service and its impact on health, limited knowledge of the resources available to veterans [33], and information and communication challenges between VA and non-VA care providers [33, 34]. Veteran care preferences are often related to geographic distance to care, relationship with a provider, cost, and perceived quality of care [5]. Care that lacks coordination, whether caused by dual use of insurance or health systems, records sharing barriers, or administrative burdens impacts veterans’ satisfaction and care outcomes [8]. Integrating these challenges into a systems model allows for a more comprehensive understanding of how VA policies, individual veterans’ needs, clinic characteristics, and the broader political context interact to produce disparities in health care access for rural veterans.

### Advantages and limitations of modeling approach

Due to restrictions on in-person meetings during the COVID-19 pandemic, we decided to use a modified participatory process in lieu of standard group modeling sessions. This change made out of necessity resulted in several modeling advantages. Because qualitative data were carefully abstracted into the model using an approach developed by Kim and Andersen [35] and subsequent researchers [17, 21, 36–39], initial participants could share their perspective in an interview, which is likely a more familiar and convenient format for many stakeholders than a group modeling session. Abstracting causal information from individual qualitative interviews is time consuming, but is the most thorough way of gleaning models from qualitative data [17]. Using summaries of some interviews to inform the model saved time and enabled us to leverage prior qualitative analysis conducted by our study team. Relying on secondary analysis of qualitative data to represent some participants’ perspectives did, however, present some limitations. Because the interviews were not originally conducted with modeling in mind, the amount of relevant causal information was limited to what emerged through the standard qualitative interview format. Interviews designed to elicit information about causal structure might have generated more data for modeling [17, 40, 41]. Moreover, involving more stakeholders in the participatory sessions would have allowed for more robust model validation.

Developing a draft model prior to engaging the stakeholder group in live sessions allowed us to minimize the amount of synchronous meeting time used, which enabled participation from busy clinician stakeholders. Group modeling typically requires significant synchronous meeting time and requires participants to engage in a new type of activity to share their perspective [21]. When the development of a shared understanding among a specific group of people is not a top priority in a modeling project, as in the needs assessment we conducted, a hybrid approach to participatory modeling may be advantageous. It is possible, however, that the virtual nature of participation constrained the quality or type of engagement by participants.

The use of a web-based platform such as Kumu for model development and communication expanded the options for displaying and viewing the model. Users can zoom and pan to model sections and click on individual variables and loops for more information about specific model elements. User controls also enable selective display of variables and causal links by stakeholder source. The web-based platform also allowed us to develop a walkthrough presentation in which model segments were accompanied by text. The walkthrough was shared with other stakeholders and researchers following the participatory sessions. While the use of web-based platforms expanded the possibilities for engaging users in the model, it also requires some expertise to develop.

Finally, findings in participatory modeling are shaped by the perspectives of the participants and the modelers involved in the process [18]. We sought to maximize transparency by using a systematic process of identifying causal information in qualitative data and by documenting source type at the level of individual causal links in the web-based version of the model. Nevertheless, because this study involved a relatively small number of participants across a three-state region, findings should be considered preliminary and not necessarily generalizable across all of VISN 20 or the US more broadly.

### Systems modeling to aid intervention planning

Systems mapping and modeling approaches have a long history of being used for illustrating complex dynamics underlying social systems and identifying potential leverage points for change [15, 18, 29, 42, 43]. While the need for systems approaches to intervention planning and implementation has been identified [17, 44–47], little guidance exists for utilizing and adapting established systems approaches for this purpose. Utilizing Meadows’s framework allowed us to categorize interventions according to potential leverage, but strategies for more systematically analyzing causal-loop diagrams to identify potential leverage points and associated interventions are needed. Moreover, guidance is needed to align leverage points with targets and resources at levels feasible for researchers or community-based teams to pursue.

Opportunity also exists for integrating systems mapping and modeling into established frameworks and processes for intervention planning and implementation, particularly those that use visual diagrams, logic models, or theories of change [48, 49]. Such diagrams or models are typically linear or categorical, and are limited in their ability to communicate causal mechanisms [50, 51]. Using a causal-loop diagram alongside a more static diagram might enable a more dynamic understanding of how interventions interact with contextual factors to produce outcomes and would provide communication tools of varying degrees of complexity. Due to its inclusion of logic models of the problem and logic models of change, intervention mapping [52] could be adapted to include systems mapping and modeling. More precision in matching and adapting interventions and implementation strategies to local contexts has the potential to more efficiently utilize limited resources and ultimately improve clinical care.

### Future research

The findings of this study are currently being used to inform development of a multilevel intervention to improve rural veteran access to care in a pilot study supported by the VA Office of Rural Health. Future modeling research could include obtaining feedback from more veterans and other stakeholders to explore generalizability of this model across more of VISN 20 and to other regions in the US. Modeling could additionally be used to refine and scale-up the intervention informed by this preliminary model. Research is also needed to further develop best practices for engaging participants in modeling and for matching engagement type (e.g., standard group modeling, hybrid interview and synchronous format, or all interviews) with study or project needs. Moreover, the use of systems mapping and modeling approaches for intervention and implementation planning requires further study.

## Conclusion

Using a participatory modeling process involving stakeholders through semi-structured qualitative interviews and live stakeholder sessions, we developed a causal-loop diagram describing rural veteran access to primary care in the Northwest region of the US. The model illustrated challenges at the patient, clinic, and system level as experienced by veterans, non-VA clinicians and staff, VA clinicians and staff, and VSOs. We used the model and Meadows’s framework to identify and categorize potential interventions that could help to improve rural veteran access to care. Participatory modeling approaches utilizing both individual and group participation have the potential of expanding involvement and providing more options for stakeholder-driven modeling.

## Supplementary Information

**Additional file 1. **Model changes during participatory validation. New variables and connections added during the participatory validation process are highlighted in dark green, and variables for which more explanatory information was attached in the web-based model are highlighted in light green. These annotations highlight portions of the diagram in Figure 4. Arrows indicate hypothesized causal relationships in stakeholder mental models as gleaned from secondary analysis of semi-structured qualitative interviews and participatory modeling sessions. Blue arrows have a positive valence, while red arrows have a negative valence.

## Data Availability

To protect the identity of interview participants, we cannot provide raw qualitative data used to inform the modeling described in this study. An interactive, web-based version of our model has been made publicly accessible alongside publication at https://ekenzie.kumu.io/caravan-rural-veteran-access-to-primary-care-stakeholder-interviews-causal-loop-diagram.

## Abbreviations

MISSION Act: Maintaining Internal Systems and Strengthening Integrated Outside Networks Act
VA: Veterans Administration
VSO: Veteran Service Officer

## Footnotes

1 https://ekenzie.kumu.io/caravan-rural-veteran-access-to-primary-care-stakeholder-interviews-causal-loop-diagram
