# Supplementary material for: Long drives and red tape: mapping rural veteran access to primary care using causal-loop diagramming
Source: BMC Health Serv Res. 2022 Aug 23;22:1075. doi: 10.1186/s12913-022-08318-2 (PMC9396592; doi:10.1186/s12913-022-08318-2)
Supplement: Supplementary file 1 — Additional file 1. Model changes during participatory validation. New variables and connections added during the participatory validation process are highlighted in dark green, and variables for which more explanatory information was attached in the web-based model are highlighted in light green. These annotations highlight portions of the diagram in Figure 4. Arrows indicate hypothesized causal relationships in stakeholder mental models as gleaned from secondary analysis of semi-structured qualitative interviews and participatory modeling sessions. Blue arrows have a positive valence, while red arrows have a negative valence. [file 12913_2022_8318_MOESM1_ESM.docx]

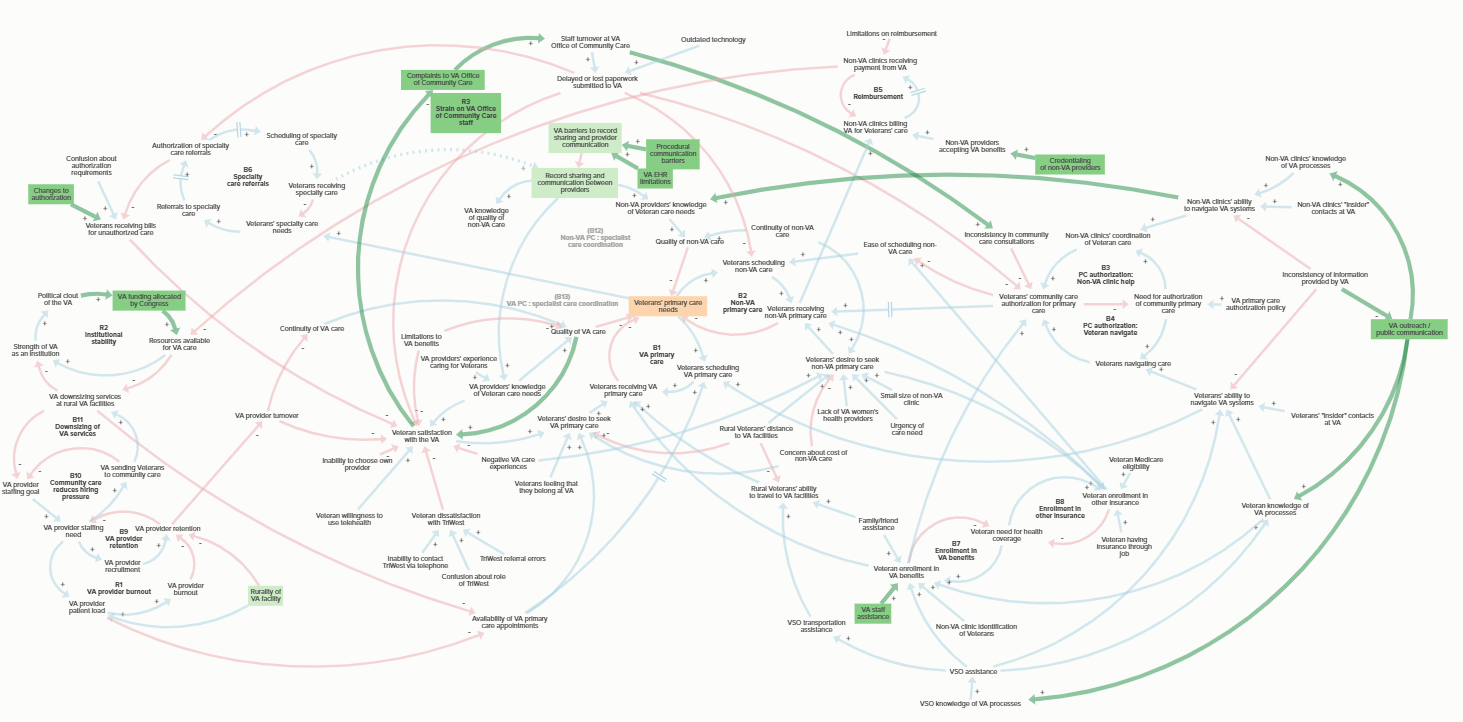


**Appendix A: Model changes during participatory validation.** New variables and connections added during the participatory validation process are highlighted in dark green, and variables for which more explanatory information was attached in the web-based model are highlighted in light green. These annotations highlight portions of the diagram in Figure 4. Arrows indicate hypothesized causal relationships in stakeholder mental models as gleaned from secondary analysis of semi-structured qualitative interviews and participatory modeling sessions. Blue arrows have a positive valence, while red arrows have a negative valence.
